# Kritischer Blick auf Einwegendoskope

**DOI:** 10.1007/s00120-025-02694-3

**Published:** 2025-10-02

**Authors:** Etienne Xavier Keller, Pascal Oechslin

**Affiliations:** 1 Department of Urology, University Hospital Zürich, University of Zürich, Frauenklinikstraße 10, 8091 Zürich, Schweiz; 2 Progressive Endourological Association for Research and Leading Solutions (PEARLS), Paris, Frankreich; 3 European Association of Urology, Section of Endourology, Arnhem, Niederlande; 4 Young Academic Urologists (YAU), Endourology & Urolithiasis Working Group, Arnhem, Niederlande

**Keywords:** Mehrwegendoskope, Recycling, Lebenszyklusanalyse, Nachhaltigkeit, Treibhausgas, Reusable scopes, Recycling, Life cycle analysis, Sustainability, Greenhouse gases

## Abstract

**Hintergrund:**

Einwegendoskope haben sich in der Urologie breit etabliert. Befürwortet werden sie dank rascher Integration technologischer Fortschritte, der Möglichkeit, Aufbereitungsanforderungen zu umgehen und dem Potenzial, Verfügbarkeitslücken zu schließen. Gleichzeitig rückt ihre ökologische Bewertung im Kontext von Nachhaltigkeit stärker in den Fokus.

**Fragestellung:**

Der Beitrag vergleicht Chancen und Nachteile von Einwegendoskop- gegenüber Mehrwegendoskopsystemen mit Schwerpunkt auf Nachhaltigkeit und Ressourcenverbrauch.

**Material und Methoden:**

Grundlagenarbeiten und Expertenempfehlungen sowie Vergleiche mit Daten aus nicht-medizinischen Quellen zu Energie- und Materialverbrauch und Abfallmanagement werden diskutiert.

**Ergebnisse:**

Herstellung, Verpackung und Distribution von Einwegendoskopen erfordern erhebliche Ressourcen, da neben Plastikelementen auch Elektronikkomponenten, Bildsensoren und Sterilisationsprozesse anfallen. Wiederverwendbare Systeme verursachen hingegen einen deutlich höheren Verbrauch von Wasser und Chemikalien in der Aufbereitung. Daten aus anderen Branchen verdeutlichen die Bedeutung geschlossener Kreisläufe und Recyclingstrategien, die bei Einwegendoskopen noch kaum etabliert sind.

**Schlussfolgerungen:**

Die aktuelle Datenlage deutet darauf hin, dass Einwegendoskope im Vergleich zu Mehrwegendoskopen eine zusätzliche Umweltbelastung verursachen könnten. Dennoch lassen die Daten derzeit keine abschließenden Folgerungen zu. Es braucht weitere Analysen, die den Ressourcen- und Energieeinsatz ganzheitlich erfassen. Recyclingstrategien und die Wiederverwendung besonders ressourcenintensiver Bauteile sind vielversprechende Ansätze für einen schonenderen Einsatz.

## Abstract (en)

**Background:**

Single-use endoscopes have become widely established in urology. They are advocated due to the rapid integration of technological advances, the possibility of bypassing reprocessing requirements, and their potential to address gaps in availability. At the same time, their ecological impact in the context of sustainability is receiving increasing attention.

**Objective:**

This article critically compares the advantages and disadvantages of single-use endoscopes versus reusable systems, with a particular focus on sustainability and resource consumption.

**Materials and methods:**

Discussion of fundamental studies and expert recommendations, supplemented by comparisons with data from nonmedical sources on energy and material use as well as waste management.

**Results:**

Production, packaging, and distribution of single-use endoscopes require considerable resources, since not only plastic elements but also electronic components, image sensors, and sterilization processes are involved. In contrast, reusable systems entail significantly higher consumption of water and chemicals during reprocessing. Evidence from other industries highlights the importance of closed-loop systems and recycling strategies, which are still scarcely implemented for single-use endoscopes.

**Conclusion:**

Current data suggest that single-use endoscopes may have a worse impact on the environment compared to reusable endoscopes. However, current data do not yet allow for definitive conclusions. Further analyses are required to comprehensively assess resource and energy demands. Introduction of recycling strategies and reuse of particularly resource-intensive components represent promising approaches towards a more sustainable application of single-use endoscopes.

## Einführung

Die ökologischen Auswirkungen von Einwegendoskopen in der Urologie rücken zunehmend in den Fokus, da Gesundheitssysteme bestrebt sind, Infektionskontrolle, Prozesseffizienz und ökologische Nachhaltigkeit auszubalancieren.

Evidenz aus systematischen Übersichten und Lebenszyklusanalysen zeigt, dass klinische Ergebnisse von Einweg- und Mehrwegendoskopen, ohne signifikante Unterschiede in Infektions- oder Komplikationsraten, vergleichbar sind [1–4]. Ob Einwegendoskope einen höheren prozedurbezogenen CO_2_-Fußabdruck verursachen, bleibt umstritten. Wesentliche Unsicherheiten bestehen fort: Variabilität von Aufbereitungsprotokollen, Gerätelebensdauer und Abfallmanagement erschweren direkte Vergleiche und limitieren die Generalisierbarkeit. Viele Studien basieren auf Einzentrumsdaten oder modellierten Szenarien, während methodische Unterschiede in Lebenszyklusanalysen (z. B. Umfang der Analyse, Emissionsfaktoren) die Robustheit der Ergebnisse beeinflussen [5]. Daten zu nachgelagerten Abfalleffekten und zur langfristigen Nachhaltigkeit hybrider Konzepte sind begrenzt und stammen aus der Gastroenterologie [6].

Dieser Beitrag bietet eine kritische Analyse der aktuellen Literatur mit besonderem Augenmerk auf methodische Qualität, um Stärken, Limitationen und Forschungsschwerpunkte für eine ökologisch tragfähige urologische Praxis zu identifizieren.

## Klinische Vor- und Nachteile von Einweg- und Mehrwegendoskopen

Die Tab. 1 bietet einen Überblick über die Vor- und Nachteile von Einweg- und Mehrwegendoskopen im klinischen Alltag. Der Hauptvorteil von Einwegendoskopen liegt darin, dass sie bei einem verbrauchsgerechten Lager in der Regel jederzeit verfügbar sind. Lediglich Unterbrechungen in der Versorgungskette könnten die Verfügbarkeit einschränken. Mehrwegendoskope hingegen erfordern eine initiale finanzielle Investition, die ihre Verfügbarkeit im klinischen Alltag bestimmt: Je geringer die Anzahl der Endoskope im Lager, desto höher ist die Wahrscheinlichkeit eines Engpasses. Hinzu kommen wiederkehrende Reparaturprozesse, welche die Verfügbarkeit zusätzlich beeinträchtigen können.

**Tab. 1:** Klinische Vor- und Nachteile von Einweg- und Mehrwegendoskopen

	Einwegendoskope	Mehrwegendoskope
Vorteile	Verfügbarkeit	Hochwertige Verarbeitung
	Rasche Integration technologischer Fortschritte	Bildqualität (je nach Modell)
	Keine Wiederaufbereitungslogistik	–
	Keine Kontaminationsgefahr	–
Nachteile	Bildqualität (je nach Modell)	Verfügbarkeit
	Verarbeitungsmängel	Wiederaufbereitungsprozess (inklusive Kontaminationsgefahr)
Kontrovers, je nach Studie und je nach institutionellen Faktoren	Umweltbelastung	Umweltbelastung
	Kosten	Kosten

Ein weiterer markanter Vorteil von Einwegendoskopen ist die rasche Integration neuer Technologien oder Techniken. Seit der Einführung von Einwegureterorenoskopen im Jahr 2008 sind mittlerweile mindestens 17 verschiedene Einwegmodelle mit FDA- (Food & Drug Administration) und CE-Zulassung verfügbar, die sich in ihren physikalischen und technischen Eigenschaften unterscheiden: Miniaturisierung, Drucksensoren, Doppelarbeitskanal, neue Handgriffdesigns sowie Knopfsteuerungen zur aktiven Kanalabsaugung sind Beispiele aktueller Fortschritte [7–11]. Die schnelle Entwicklung der Einwegendoskope ermöglicht es Urologen, Geräte auszuwählen, die spezifisch auf klinische Szenarien und persönliche Präferenzen zugeschnitten sind. Dies unterstreicht sowohl die Agilität des Marktes als auch die Leichtigkeit, mit der neue Technologien in die klinische Praxis integriert werden können [12].

Einwegendoskope sind spezifisch auf klinische Szenarien und persönliche Präferenzen zugeschnitten

Generell lässt sich feststellen, dass die Verarbeitung von Mehrwegendoskopen qualitativ höherwertiger ist, als jene von Einwegendoskopen. Eine konisch geformte Spitze, sanft abgerundete Kanten, eine leistungsfähigere Lichtquelle mit optimierter Ausleuchtung sowie eine hochstechende Bildqualität sind exemplarische Merkmale dafür [11, 13].

Der Wiederaufbereitungsprozess stellt hingegen einen wesentlichen Nachteil von Mehrwegendoskopen dar. Neben erheblichem Ressourcenverbrauch (Wasser, Energie, Chemikalien, Verbrauchs- und Verpackungsmaterial) erfordern Aufbau, Zertifizierung und Instandhaltung einer Aufbereitungseinheit sowie die Bindung an qualifiziertes Personal beträchtliche finanzielle und räumliche Mittel, die für kleinere Institutionen oft nicht mehr tragbar sind. Hinzu kommt das potenzielle Risiko einer mikrobiellen Kontamination infolge unzureichender Reinigung; dieses lässt sich jedoch bei konsequenter Einhaltung und Überwachung einschlägiger Normen auf ein sehr niedriges Niveau reduzieren [14].

## Nachhaltigkeit: Evidenz aus der urologischen Literatur

Acht Studien der aktuellen urologischen Fachliteratur verglichen den ökologischen Fußabdruck von Einweg- und Mehrwegendoskopen [15–22], davon 2 Studien mit Ureterorenoskopen [15, 16] und 6 mit Zystoskopen [17–22]. Der primäre Endpunkt aller Studien war das Treibhausgaspotenzial (kg CO_2_-Äquivalent) pro Anwendung. Darüber hinaus wurden in einer Studie [19] zusätzliche Umweltindikatoren wie Ressourcenverbrauch (Megajoule), Ökotoxizität (kg 1,4-Dichlorbenzol-Äquivalent), Versauerungspotenzial (kg SO_2_-Äquivalent) und Eutrophierungspotenzial (kg PO_4_-Äquivalent) berechnet. Eine weitere Studie [16] berücksichtigte zusätzlich die Gesundheitsauswirkungen in Form von „disability-adjusted life-years“ (DALY), basierend auf dem Treibhausgaspotenzial.

Die Tab. 2 fasst die Methodologie und die Ergebnisse der einzelnen Vergleichsstudien zusammen. Am häufigsten untersucht wurde das Einwegendoskop Ambu® aScope™ 4 Cysto (Ballerup, Dänemark; 5 von 8 Studien; [17–21]), gefolgt von jeweils einer Studie mit Boston Scientific LithoVue (Boston, MA, USA; [15]) und Coloplast Isiris (Humlebæk, Dänemark; [22]); in einer Studie wurde das Einwegendoskop nicht näher spezifiziert.

**Tab. 2:** Methodologie und Ergebnisse von Vergleichsstudien über Einweg- und Mehrwegendoskope

Autor, Jahr, Referenz	Beteiligung des Herstellers	Einwegendoskope	Mehrwegendoskope	Treibhausgasemissionen (kg CO_2_-Äq.)*		Ratio Einweg/Mehrweg	Nachhaltigkeitsvorteil
				Einweg	Mehrweg		
*Ureterorenoskope*							
Davis, 2018 [15]	Nein	Boston Scientific LithoVue	Olympus URV‑F	4,43	4,47	0,99	Einwegendoskop
Thöne, 2024 [16]	Ja (Methodologie)	n.v.	n.v.	4,93	1,24	3,98	Mehrwegendoskop
*Zystoskope*							
Boucheron, 2022 [17]	Nein	Ambu aScopeTM 4 Cysto	n.v.	n.v.	n.v.	n.v.	Einwegendoskop
Hogan, 2022 [18]	Ja (Methodologie)	Ambu aScopeTM 4 Cysto	Olympus CYF-VA2	2,41	4,23	0,57	Einwegendoskop
Baboudjian, 2023 [19]	Ja (Finanzierung und Methodologie)	Ambu aScopeTM 4 Cysto	n.v.	2,06	3,08	0,67	Einwegendoskop
Kemble, 2023 [20]	Ja (Methodologie)	Ambu aScopeTM 4 Cysto	Olympus CYF-V2	2,54	0,53–1,04	2,44–4,8	Mehrwegendoskop
Wombwell, 2023 [21]	Ja (Methodologie)	Ambu aScopeTM 4 Cysto	Olympus CYF-VH	1,43	2,22	0,64	Einwegendoskop
Jahrreiss, 2024 [22]	Ja (Methodologie)	Coloplast Isiris	Keines. Hypothetische Berechnung basierend auf Davis et al., angepasst für Zystoskope	1,76	3,95–4,47	0,39–0,45	Einwegendoskop

*n.v*. nicht vorhanden, *Äq.* Äquivalent

* pro Anwendung

Die Treibhausgasemissionen pro Verwendung zeigten deutliche Unterschiede: bei Einwegureterorenoskopen 4,43–4,93 kg CO_2_-Äquivalent, bei Mehrwegureterorenoskopen 1,24–4,47 kg CO_2_-Äquivalent; bei Einwegzystoskopen 1,43–2,54 kg CO_2_-Äquivalent und bei Mehrwegzystoskopen 0,53–4,47 kg CO_2_-Äquivalent.

Bei der Ureterorenoskopie liegt der Nachhaltigkeitsvorteil klar zugunsten von Mehrwegendoskopen

Ein Nachhaltigkeitsvorteil zugunsten von Einwegendoskopen liess sich in 6 von 8 Studien feststellen, mit einem Treibhausgasreduktionspotenzial von 0,01- bis 2,6-fach im Vergleich zu Mehrwegendoskopen. Zwei Studien zeigten hingegen einen Vorteil für Mehrwegendoskope, mit einem 2,44- bis 4,8-fachen Reduktionspotenzial.

Die bisher umfassendste veröffentlichte Lebenszyklusanalyse [16] deutet darauf hin, dass bei der Ureterorenoskopie der Nachhaltigkeitsvorteil klar zugunsten von Mehrwegendoskopen liegt. Selbst unter Berücksichtigung unterschiedlicher Transportwege vom Hersteller zum Spital und variierender Stromversorgungsmodelle bleibt dieser Vorteil bestehen. Bemerkenswert ist, dass in der Studie von Thöne et al. [16] Reparaturen von Mehrwegendoskopen den Nachhaltigkeitsvorteil erhalten, selbst wenn bei jeder Anwendung eine Reparatur erforderlich wäre. Ein signifikanter Vorteil für Mehrwegendoskope zeigte sich bereits bei einer Nutzung von mehr als sieben Anwendungen vor Erreichen der geplanten Lebensdauer.

## Qualität der Evidenz und methodischen Überlegungen

Das Treibhausgaspotenzial (kg CO_2_-Äquivalent) wurde in allen oben genannten Studien auf Basis von Lebenszyklusanalysen (LCA) berechnet. Gemäß ISO 14040/14044-Standard stellt die LCA eine systematische Methode zur Erfassung und Bewertung der Umweltaspekte und potenziellen Umweltauswirkungen eines Produktsystems über dessen gesamten Lebenszyklus dar – von der Rohstoffgewinnung über Produktion und Nutzung bis zur Entsorgung [23, 24].

**Abb. 1:**
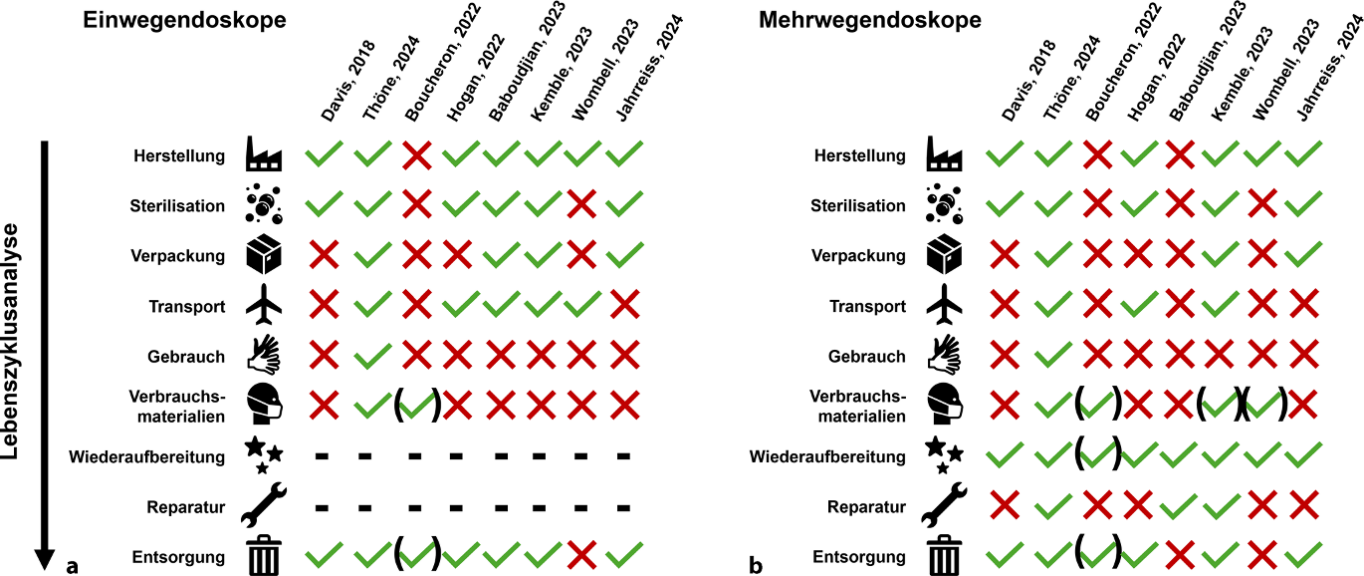
Umfang der Lebenszyklusanalyse aus den Vergleichsstudien. *Grüne Häkchen in Klammern* bedeutet, dass der Umfang der Analyse im gekennzeichneten Bereich methodisch nicht umfassend war

Die Abb. 1 zeigt eine detaillierte Auswertung des Umfangs der LCA der zuvor genannten Vergleichsstudien, basierend auf den methodischen Angaben aus den Originalpublikationen [15–22]. Während einige Studien die LCA auf die Messung des rohen Abfallgewichts oder den Wasserverbrauch bei der Wiederaufbereitung beschränkten [17], beinhaltet die LCA in der Studie von Thöne et al. [16] eine umfassende Analyse vom Herstellungsprozess bis zur Entsorgung, inklusive Berücksichtigung der im Wiederaufbereitungsprozess verwendeten Chemikalien und Verbrauchsmaterialien.

Aus Abb. 1 lässt sich ableiten, dass die meisten Studien bei Einwegendoskopen den Schwerpunkt auf Herstellung und Entsorgung legten, während bei Mehrwegendoskopen der Fokus auf dem Wiederaufbereitungsprozess lag. Diese Vorgehensweise ist nachvollziehbar: Bei Einwegendoskopen entfallen Wiederaufbereitungsprozesse und Reparaturen. Bei Mehrwegendoskopen wird der ökologische Fußabdruck aus Herstellung, Reparaturen und Entsorgung durch die Anzahl Wiederverwendungen (Lebenszyklen) geteilt – durch einen Faktor von 133 [16] bis 180 [15] bei Ureterorenoskopen und von 1120 [18] bis 3920 [20, 21] bei Zystoskopen. Daraus schließen die Autoren mehrerer Studien, dass das Treibhausgaspotenzial aus Herstellung, Reparaturen und Entsorgung von Mehrwegendoskopen zwar auf hypothetischen Daten basiert, jedoch nur einen Bruchteil der Gesamtbilanz ausmacht und daher nur ein geringes Risiko für signifikante Abweichungen von der Realität besteht. Dennoch trägt die breite Heterogenität der LCA zwischen den Studien zur Komplexität der Interpretation bei.

Die breite Heterogenität der LCA zwischen den Studien trägt zur Komplexität der Interpretation bei

Bezüglich Einwegendoskopen ist hervorzuheben, dass die Berechnung des Treibhausgaspotenzials aus dem Herstellungsprozess in nahezu allen Studien auf Herstellerangaben beruhte und in keiner Studie unabhängig überprüft wurde [15, 16, 18–22]. Die anteilsmäßigen Angaben (prozentual pro Endoskop) variierten leicht zwischen den Studien und sind in Tab. 3 zusammengefasst. Bemerkenswert ist, dass in allen Studien dieselben Referenzen für die Berechnung der Treibhausgasäquivalente pro Materialkomponente verwendet wurden, meist aus nicht überprüften Internetquellen: Kunststoff 6 kg CO_2_-Äquivalent [25], Gummi 1,16 kg CO_2_-Äquivalent [26], Stahl 1,8 kg CO_2_-Äquivalent [27] und Elektronik 150 kg CO_2_-Äquivalent [28]. Zwei Ausnahmen bilden die Studien von Baboudjian et al. [19] und Thöne et al. [16], welche die Ecoinvent-Datenbanken v3.5 bzw. v3.8 nutzten, aber allerdings weiterhin auf Herstellerangaben zur Zusammensetzung der Endoskope zurückgriffen.

**Tab. 3:** Anteilsmäßiger Beitrag einzelner Komponenten aus dem Herstellungsverfahren

		Basierend auf Ecoinvent v3.5 rsp. v3.8 [16, 19]		Andere Studien [15, 18, 20–22]	
Komponente	Anteil basierend auf Endoskop Rohgewicht (%) [15, 16, 18–22]	kg CO_2_-Äq. pro kg Komponente	Anteil Treibhausgas (kg CO_2_-Äq) [%]	kg CO_2_-Äq. pro kg Komponente	Anteil Treibhausgas (kg CO_2_-Äq) [%]
Kunststoff	90–95,3	3,4	79	6	47–67
Gummi	0,4–3,31	n.v.	n.v.	1,16	0,2
Stahl	4–9	9,1	21	1,8	0,5–0,6
Elektronik	0,1–4	120	0,2	150	32–52

*Äq.* Äquivalent,* n.v.* nicht vorhanden

Die Treibhausgasberechnung aus dem Herstellungsprozess beruhte in nahezu allen Studien auf Herstellerangaben

Die Daten aus Tab. 3 deuten darauf hin, dass der Kunststoffanteil maßgeblich für das Treibhausgaspotenzial von Einwegendoskopen ist. Bei der genaueren Analyse des Elektronikanteils lassen sich zusätzlich zwei wichtige Beobachtungen machen: Erstens bestehen Diskrepanzen zwischen den angegebenen Elektronikgewichten, welche in einem Endoskop enthalten sind – 0,2 g in der Studie von Thöne et al. (Ureterorenoskop; [16]) vs. 12 g (60-fach mehr) in der Studie von Davis et al. (Zystoskop; [15]). Angesichts des hohen Treibhausgaspotenzials der Elektronik (120–150 kg CO_2_-Äquivalent gemäß analysierten Studien) sollte der tatsächliche Beitrag der Elektronik in zukünftigen Studien unabhängig überprüft und kritisch hinterfragt werden. Zweitens gilt es hervorzuheben, dass eine Übersichtsarbeit über LCA von Smartphones und Tablets aus dem Jahr 2020 eine große Variabilität des Treibhausgaspotenzials im Herstellungsprozess zeigte, wobei die Elektronik jeweils den größten Anteil ausmachte [29].

Es sind zukünftig Studien erforderlich, die den Beitrag der Elektronik aufschlüsseln

Eine LCA des Smartphones „Fairphone 5“ (Fairphone B.V., Amsterdam, Niederland) aus dem Jahr 2024 zeigt, dass allein die Kameramodule ein erhebliches Treibhausgaspotenzial besitzen: 2,83 kg CO_2_-Äquivalent für die Ultraweitwinkelkamera, 1,57 kg CO_2_-Äquivalent für die Hauptkamera und 1,34 kg CO_2_-Äquivalent für die Frontkamera. Da jedes Einwegendoskop mit einem Kameramodul ausgestattet ist, sind zukünftige Studien erforderlich, die den Beitrag der Elektronik aufschlüsseln und unabhängig von Herstellerangaben detailliert beziffern. Letztlich bleibt zu klären, ob etwa Elektronik – und nicht Kunststoff – maßgebend für das Treibhausgaspotential aus dem Herstellungsprozess von Endoskopen ist.

## Daten aus nicht-urologischer Fachliteratur

Studien aus der Gastroenterologie haben mehrheitlich einen Nachhaltigkeitsvorteil zugunsten von Mehrwegendoskope gezeigt. Ein kürzlich publizierte LCA von Gastroskopen zeigte, dass Einweggeräte pro Untersuchung einen 2,5-fach höheren CO_2_-Fußabdruck verursachen als Mehrwegendoskope Geräte (10,9 kg vs. 4.7 kg CO_2_-Äquivalent; [30]). Ähnlich verhält es sich im Zusammenhang mit Duodenoskopen: Einweggeräte waren pro Untersuchung mit 24- bis 47-mal höheren Treibhausgasemissionen verbunden als Mehrwegendoskope Duodenoskope, wobei der größte Teil der Auswirkungen auf die Herstellung zurückzuführen war [31]. Abfallaudits in Endoskopiezentren mit hohen Fallzahlen aus den USA haben gezeigt, dass ein vollständiger Umstieg auf Einwegendoskope die Nettoabfallmenge im Vergleich zur Mehrwegendoskop um etwa 40 % erhöhen würde – selbst unter Berücksichtigung des Abfalls, der durch die Aufbereitung mehrwegendoskoper Geräte entsteht [6]. Eine systematische Übersichtsarbeit berichtete übereinstimmend, dass hochwertige LCA Mehrwegendoskope im Hinblick auf Nachhaltigkeit begünstigen, auch wenn die Ergebnisse je nach lokalen Aufbereitungspraktiken und Gerätetyp variieren können [32]. Die Amerikanische Gesellschaft für Gastrointestinale Endoscopie hat ebenfalls hervorgehoben, dass eine weitverbreitete Einführung von Einwegendoskopen erhebliche Umweltschäden verursachen würde, mit deutlich erhöhtem CO_2_-Fußabdruck und gesteigerter Abfallmenge im Vergleich zu Mehrwegendoskopgeräten [33]. Wohlbemerkt sind gastroenterologische Endoskope nicht gänzlich mit Ureterorenoskopen oder Zystoskopen gleichzustellen. Deren Aufbau und Anforderungen differenzieren insofern, dass ein vergleichsweiser zusätzlicher Aufwand bei Gasteroenteroskopen in der Herstellung anfällt (mehr Arbeitskanäle und Anschlüsse, robusterer Aufbau). Dennoch signalisiert dieser Vergleich, dass die Umweltbilanz von Endoskopen in der Urologie weiter geprüft und analysiert werden sollte.

## Aussichten: Geschlossene Kreisläufe und Recyclingstrategien

Die Wiederverwendung von Einwegendoskopen ist – wie bereits die Bezeichnung nahelegt – unzulässig, da deren Konstruktion die Einhaltung standardisierter Wiederaufbereitungsprozesse verunmöglicht. Dennoch existieren Berichte über diese Praxis, die in der Regel primär finanziell motiviert ist und weniger aus ökologischen Überlegungen erfolgt [34].

Bislang bietet kein Hersteller von Einwegendoskopen etablierte und verifizierte Recyclingstrategien an. Eine künftige Evaluation der Wiederverwendung besonders ressourcenintensiver Bauteile, etwa des Kamerakopfes, wäre jedoch wünschenswert.

Ein hybrides Konzept stellt das EndoSheath™-Zystoskop (Vision Sciences, Orangeburg, NY, USA) dar: Hierbei wird ein transparenter Einwegschaft über den semizirkulären Schaft eines Mehrwegzystoskops gezogen, sodass nach der Intervention lediglich der Einwegschaft entsorgt wird [35]. Der wiederverwendbare Teil des Zystoskops bedarf keiner hochgradigen Desinfektion, womit sich Treibhausgasemissionen des Wiederaufbereitungsprozesses reduzieren lassen. Abgesehen von Berichten über eine leicht erschwerte Handhabung und eine etwas eingeschränkte Bildqualität belegen mehrere Studien ein hohes Sicherheitsprofil ohne Hinweis auf vermehrte Infektkomplikationen [36–38].

## Schlussfolgerungen

Die aktuelle Datenlage deutet darauf hin, dass Einwegendoskope im Vergleich zu Mehrwegendoskopen eine zusätzliche Umweltbelastung verursachen könnten. Dennoch lassen die Daten derzeit keine abschließenden Folgerungen zu.

Recyclingstrategien besonders ressourcenintensiver Bauteile sind vielversprechend

Die unterschiedlichen Resultate zwischen den Studien lassen sich nur teilweise durch lokale Bedürfnisse und Abläufe erklären. Vielmehr scheint die Methodologie der Studien einen maßgebenden Einfluss auf die Berechnungen des Treibhausgaspotenzials von Endoskopen auszuüben. Es bedarf weiterer Analysen, die Ressourcen- und Energieeinsatz ganzheitlich erfassen und auf verifizierten, unabhängigen Daten basieren. Recyclingstrategien sowie die Wiederverwendung besonders ressourcenintensiver Bauteile stellen vielversprechende Ansätze für einen nachhaltigeren Einsatz dar.

## Fazit für die Praxis

- Einwegendoskope bieten den Vorteil, bei einem verbrauchsgerechten Lager in der Regel jederzeit verfügbar zu sein.
- Ein weiterer markanter Vorteil von Einwegendoskopen ist die rasche Integration neuer Technologien oder Techniken.
- Der Wiederaufbereitungsprozess stellt einen wesentlichen Nachteil von Mehrwegendoskopen dar.
- Lebenszyklusanalysen aus 8 Vergleichsstudien zeigen uneinheitliche Ergebnisse zu Treibhausgasemissionen.
- Eine veröffentlichte Lebenszyklusanalyse deutet darauf hin, dass bei der Ureterorenoskopie der Nachhaltigkeitsvorteil klar zugunsten von Mehrwegendoskopen liegt.
- Studien aus der Gastroenterologie haben mehrheitlich einen Nachhaltigkeitsvorteil zugunsten von Mehrwegendoskope gezeigt.
- Anteilsmäßig bleibt zu klären, welche Komponenten bei Einwegendoskope am meisten zum Treibhausgaspotential bei der Herstellung von Endoskopen beiträgt: Plastik, Gummi, Stahl oder Elektronik.
- Geschlossene Kreisläufe und Recyclingstrategien bieten die Möglichkeit, zukünftig die ökologische Bilanz von Einwegendoskopen zu verbessern.

## Data Availability

Daten, die aus den einbezogenen Studien extrahiert wurden, können auf begründete Anfrage beim korrespondierenden Autor bereitgestellt werden.
